# The determination of Ochratoxin A based on the electrochemical aptasensor by carbon aerogels and methylene blue assisted signal amplification

**DOI:** 10.1186/s13065-018-0415-4

**Published:** 2018-04-25

**Authors:** Min Wei, Wenyang Zhang

**Affiliations:** 1 College of Food Science and Technology, Henan University of Technology, Zhengzhou, 450001 People’s Republic of China; 2 Henan Key Laboratory of Cereal and Oil Food Safety Inspection and Control, Henan University of Technology, Zhengzhou, 450001 People’s Republic of China

**Keywords:** Ochratoxin A, The CAs-cDNA/apt/AuE aptasensor, Carbon aerogels, Methylene blue

## Abstract

In this work, a novel aptamer-based electrochemical biosensor was developed for the determination of Ochratoxin A (OTA) by using carbon aerogels (CAs) and methylene blue (MB) as signal amplification strategy. CAs was used as carrier to load the abundant of complementary DNA (cDNA), which could enhance the hybridization between CAs-cDNA and aptamer immobilized on the electrode surface, thus provide more double-stranded DNA for MB intercalation. The current of MB on the CAs-cDNA/apt/AuE sensor was twice that on the cDNA/apt/AuE sensor, which indicated that the CAs with high surface area enabled a higher loading of the cDNA and absorbed more MB, thus realized the signal amplification strategy. The optimum experimental conditions including MB incubation time of 15 min, aptamer concentration of 4.0 μmol/L, hybridization time of 2.0 h, and OTA incubation time of 18 min were obtained. The change of peak current was linearly proportional to the OTA concentration in the range of 0.10–10 ng/mL with the actual detection limit of 1.0 × 10^−4^ ng/mL. The experimental results showed that the prepared CAs-cDNA/apt/AuE exhibited good specificity, acceptable reproducibility and repeatability. This sensor was applied to detect OTA in the spiked corn samples, and obtained an acceptable average recovery of 89%.

## Background

Mycotoxins are toxic contaminants produced by the secondary metabolism of fungi, mainly saprophytic molds [1]. As one of the highly toxic mycotoxins, Ochratoxin A (OTA) secreted by Aspergillus and Penicillium has attracted much more attention because it contaminates broad range of agricultural products such as maize, wheat, rice, coffee, and peanut, then results in serious human and animal health problems including nephrotoxic, hepatotoxic, neurotoxic, teratogenic and immunotoxic activities [2]. So, it is increasingly necessary to develop a precise, rapid and low-cost method for OTA determination in various samples. Conventional instrumental analyses such as high performance liquid chromatography, liquid chromatography tandem mass spectrometry, and fluorescence are popular because of their high sensitivity, good accuracy and reproducibility [3–6]. However, they exist some drawbacks such as sophisticated equipment, high cost and requirement of technical skills [7]. The immunoassay methods based on antigen–antibody binding have the advantages of simple, rapid and easy to operation, and appear an useful tool for on-site detection of OTA [8–11]. However, the antibody preparation process is complex and time-consuming, high cost, and the antibody itself is unstable, immunogenic and false. So it can not be used as a final confirmation method, which hinders its wider application.

As a novel bio-recognition element, aptamers, single strand oligonucleotides, with the superiority including strong affinity, high stability, and easy modification of functional groups, have the potential designing highly sensitive, selective and structure switchable sensing assays [12–14]. Recently, aptamer-based electrochemical biosensors for OTA detection are prominent owing to their fast response, low cost, simple operation, easy to miniaturization of the instrument, and portability [15–19].

To realize the signal amplification and improve the sensitivity of the electrochemical aptasensors, nanomaterials have been chosen because their large specific surface area allows immobilizing more signal molecules on the electrode surface, and their well electronic conductivity makes the charge transfer to the electrodes easier [20–23]. Due to their favorable properties including great mesopore volume, high accessible surface area and good electrical conductivity, carbon aerogels (CAs) have attracted tremendous attention and have been extensively used as supports of precious metal for electrocatalytic reaction [24, 25], whereas have seldom been used for immobilization of biomolecules [26].

In this work, a novel aptamer-based electrochemical biosensor was developed for the determination of OTA by using CAs and methylene blue (MB) as signal amplification strategy. CAs was used as carrier to load the abundant of complementary DNA (cDNA), which could enhance the hybridization between CAs-cDNA and aptamer immobilized on the electrode surface, thus provide more double-stranded DNA for MB intercalation. As an electrochemical indicator, MB could intercalate both into single-stranded cDNA through the guanine bases and into double-stranded DNA, and produce a strong current signal. When OTA existed, the formation of aptamer-OTA complex changed the conformation of aptamer and prohibited the binding of cDNA-aptamer, which resulted in the release of MB from the electrode surface and produced a reduced current signal. The change of MB current signal could be used for OTA detection.

## Methods

### Materials and chemicals

1-Ethyl-3-(3-dimethylaminopropyl)-carbodiimide (EDC), N-hydroxysuccinimide (NHS), methylene blue (MB) were purchased by Macklin Biochemical Co., Ltd. (Shanghai, China). All oligonucleotides were synthesized by Sangong Biotech (Shanghai, China) Co., Ltd., and their base sequences were: complementary DNA (cDNA): 5′-NH_2_-GGA GGA GGA GGA GGA GGA GGA GGA GGA GGA GGA GGA GGA TGT CCG ATG CTC CCT TTA CGC CTC-3′; OTA aptamer (apt): 5′-HS-GAT CGG GTG TGGGTG GCG TAA AGG GAG CAT CGG ACA-3′. 50 mM, pH7.4 Tris–HCl was prepared by 0.20 M NaCl and 1.0 mM EDTA and adjusting the pH with 0.10 M HCl. All other chemicals were of analytical-reagent grade.

### Apparatus

All the electrochemical experiments were performed on a CHI 660E Electrochemical Workstation (Shanghai Chenhua Instrument Corporation, China). A three-electrode system was comprised of Au electrode (AuE) as working electrode, platinum wire as auxiliary electrode, and Ag/AgCl as reference electrode. Scanning electron microscopy (SEM) was performed using a JEOL JSM7100F SEM facility (Jeol, Japan).

### Preparation of the CAs-cDNA/apt/AuE sensor for OTA detection

The AuE was polished with 0.30 and 0.050 μm gamma alumina powder successively and then rinsed with ultrapure water and dried by nitrogen. The AuE was activated by scanning cyclic voltammogram (CV) with 0.50 M H_2_SO_4_.

CAs were synthesized by the sol–gel polymerization of resorcinol (R) and formaldehyde (F) in an aqueous solution according to the method described elsewhere [25]. cDNA (25 μL, 100 μM) was put into 500 μL of CAs suspension, then 250 μL of EDC and NHS was separately added into the solution, the mixture was incubated overnight at 37 °C. Next, the above solution was incubated with NaCl (50 μL, 2.0 M) for 24 h and centrifugated at 12,000 rpm to remove the unbound cDNA. The precipitate was repeatedly rinsed and redispersed in 5.0 mL Tris–HCl solution to obtain the CAs-cDNA.

**Scheme 1:**
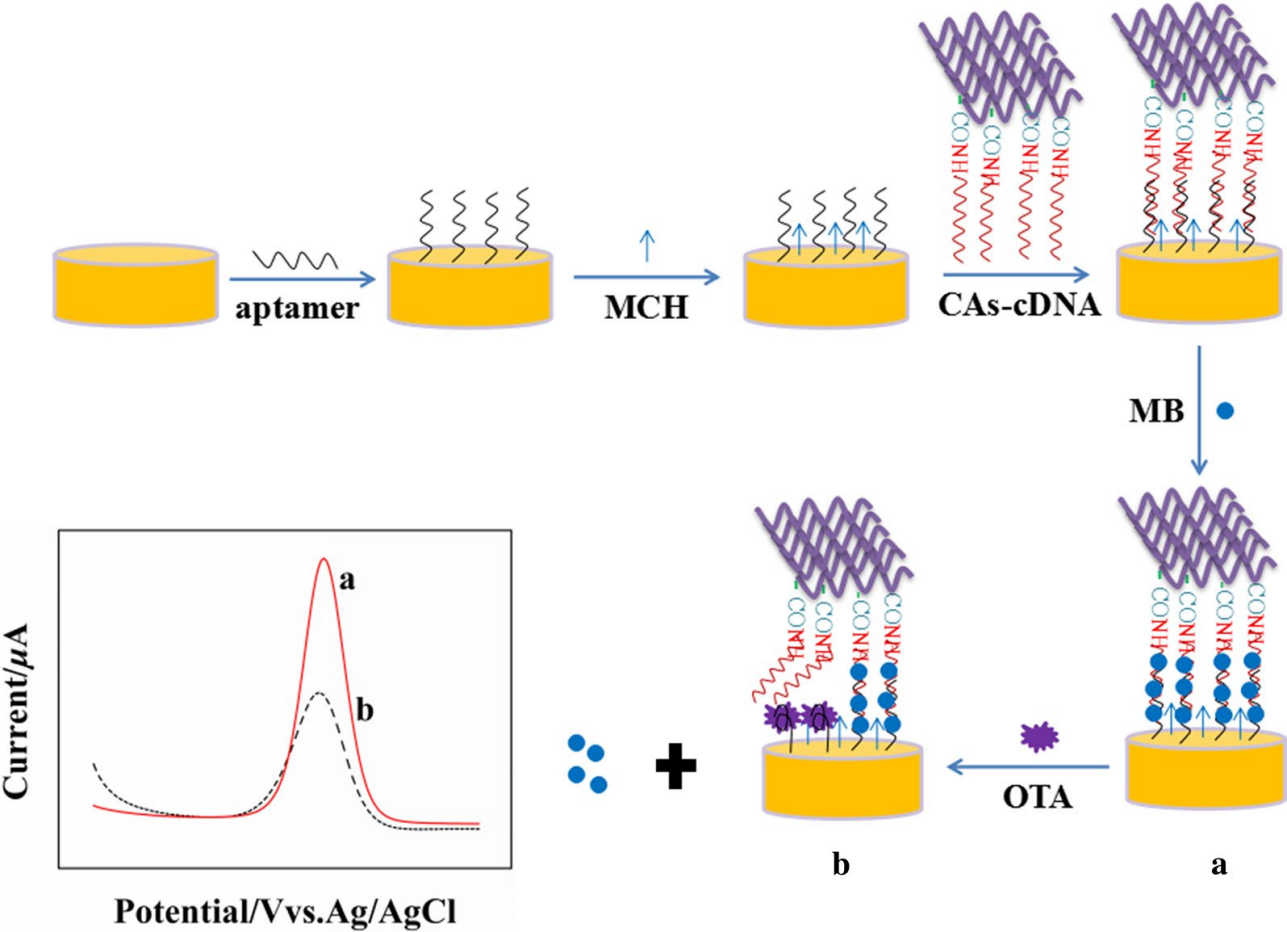
Schematic illustration of the procedure of the CAs-cDNA/apt/AuE fabrication for OTA detection

5.0 μL of aptamer was immobilized on the surface of AuE to obtain apt/AuE via the S–Au bonds, and then the modified electrode was incubated with MCH to eliminate nonspecific binding and block the remaining active groups. Sequentially, 5.0 μL of CAs-cDNA was dropped on the apt/AuE surface and the hybridization reaction was proceeded at 37 °C to obtain the CAs-cDNA/apt/AuE sensor. The procedure of the aptasensor fabrication for OTA detection was illustrated in Scheme 1.

## Results and discussion

### Characterization of the prepared CAs

**Fig. 1:**
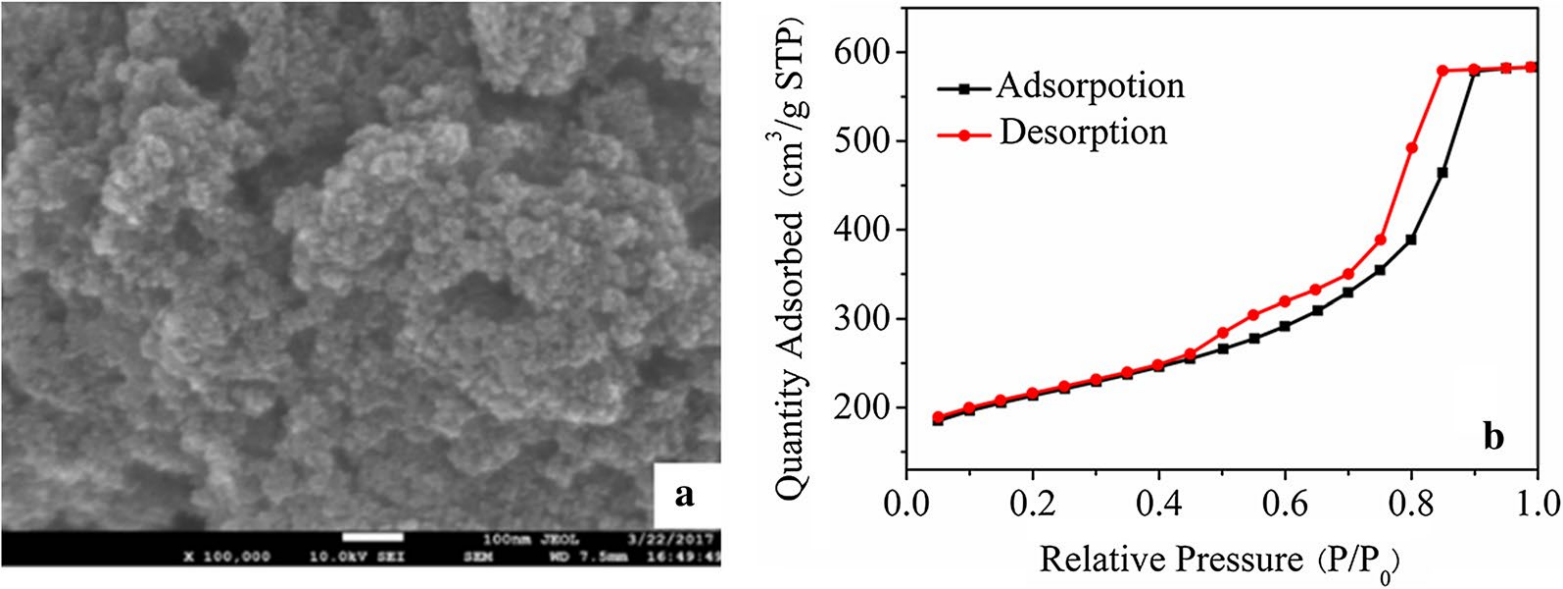
**a** SEM image and **b** N_2_ adsorption–desorption isotherms of CAs

The morphology of CAs was characterized by SEM, and the result was showed in Fig. 1a. It was observed that the prepared CAs had high surface area and three-dimensional interconnected porous structure. The sizes of particles have been found to range from 50 nm to 100 nm and uniformly distributed. The pore sizes between particles were in the range of 20–150 nm. Figure 1b showed the nitrogen adsorption–desorption isotherms of CAs. It displayed an obvious hysteresis loop, indicating the presence of mesopores in CAs. The surface area and pore volume of CAs were measured on Autosorb IQ (Quantachrome) using the Brunauer–Emmett–Teller (BET) method. The BET surface area and pore volume of CAs were 695 m^2^ g^−1^ and 0.90 cm^3^ g^−1^, respectively. These high surface area and porous structure made CAs expose more active sites, accelerate the transfer rate and improve the electrochemical performance.

### Electrochemical characterization of the CAs-cDNA/apt/AuE sensor

**Fig. 2:**
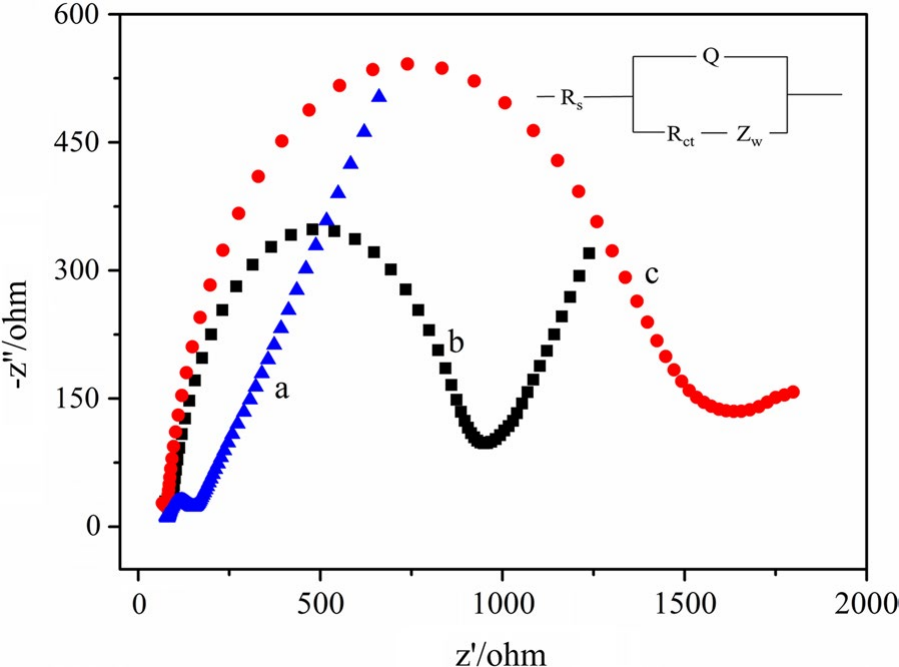
The EIS of 10.00 mM [Fe(CN)_6_]^3−/4−^ on the different electrodes. (a) The bare AuE, (b) the apt/AuE, (c) the CAs-cDNA/apt/AuE. Inset: the equivalent circuit

Electrochemical impedance spectroscopy was used to characterize the different electrodes, and the results were shown in Fig. 2. For the bare AuE (a), the charge transfer resistance (R_ct_) was 80 Ω, indicating that the bare AuE had good conductivity. When the aptamer was modified on the AuE surface to obtain the apt/AuE (b), the R_ct_ was 831 Ω, which increased obviously as compared to that of the bare AuE. This was due to that the repulsion between the negatively charged backbones of DNA strands and [Fe(CN)_6_]^3−/4−^ hindered the interfacial electron transfer. For the CAs-cDNA/apt/AuE (c), after the CAs-cDNA hybridized with the aptamer, the negative charge density of the electrode surface further increased, so the R_ct_ significantly increased to 1491 Ω. These results were also proved that the aptamer and the CAs-cDNA had been successfully immobilized on the electrode surface. On the basis of the charge transfer kinetics of the [Fe(CN)_6_]^3−/4−^, the Faradaic impedance spectra were modeled using the Randles equivalent circuit (inset of Fig. 2). The fitting parameters involved the resistance of the solution (Rs), the electron-transfer resistance (Rct), Warburg impedance (Zw) attributed to the contribution of diffusion, and the constant phase element (Q).

### Electrochemical behavior of MB on the different sensors

**Fig. 3:**
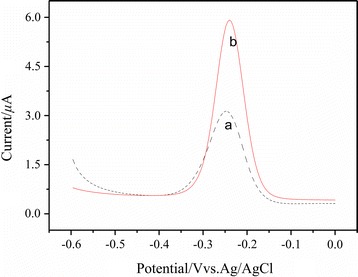
DPV responses of different electrode in Tris–HCl buffer after incubated in 0.02 μM MB. (a) The cDNA/apt/AuE, (b) the CAs-cDNA/apt/AuE

Using MB as the electrochemical probe, the cDNA/apt/AuE (a) and the CAs-cDNA/apt/AuE (b) were characterized by the differential pulse voltammetry (DPV). As shown in Fig. 3, the peak current of MB was 2.7 μA on the cDNA/apt/AuE and 5.4 μA on the CAs-cDNA/apt/AuE. The peak current on the CAs-cDNA/apt/AuE was twice that on the cDNA/apt/AuE, which was ascribed to that the high surface area and porous structure of CAs made it load more cDNA and absorb more MB, thus realize the signal amplification.

### The detection mechanism of OTA based on the CAs-cDNA/apt/AuE sensor

**Fig. 4:**
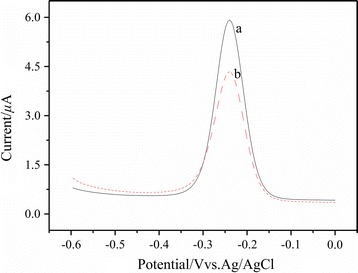
The DPV of the CAs-cDNA/apt/AuE in Tris–HCl buffer after incubated in 0.02 μM MB. (a) 0 ng/mL OTA, (b) 10 ng/mL OTA

Figure 4 showed the signal change of 0.02 μM MB before and after incubation with 10 ng/mL OTA. The peak current of MB on the CAs-cDNA/apt/AuE (a) was 5.4 μA and obviously decreased to 3.8 μA on the OTA/CAs-cDNA/apt/AuE (b). The ΔI was 1.6 μA before and after incubation with OTA, which can be applied for OTA detection. This signal change can be explained as follows: In the absence of OTA, MB can intercalate both into single-stranded cDNA through the guanine bases and into double-stranded DNA, thus produce a strong current signal. In the presence of 10 ng/mL OTA, the binding of OTA and aptamer is considerably greater than that of cDNA and aptamer, which results in the release of MB from the electrode surface and produces a reduced current signal.

### The optimization of the important factors

**Fig. 5:**
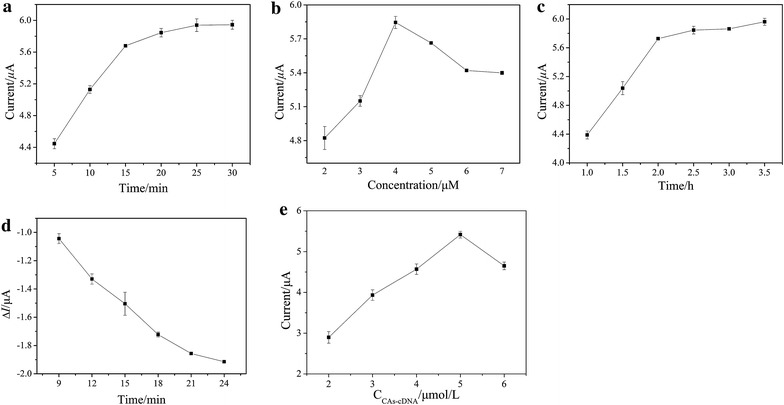
The effect of **a** the incubation time of MB, **b** the aptamer concentration, **c** the hybridization time between cDNA and aptamer, **d** the incubation time of OTA, and **e** the concentration of CAs-cDNA on the CAs-cDNA/apt/AuE sensor

The effect of incubation time of MB on the CAs-cDNA/apt/AuE sensor was studied. As shown in Fig. 5a, the peak current obviously increased with the increase of incubation time from 5 min to 15 min. The uptrend was slowly when the incubation time exceeded 15 min. So 15 min of incubation time was used in the following experimental study. Figure 5b showed the optimized results of the aptamer concentration. It can be seen that the peak current of MB obviously increased when the aptamer concentration increased from 2.0 to 4.0 μmol/L, and reached a maximum of 5.8 μA at 4 μmol/L. Beyond the aptamer concentration of 4.0 μmol/L, the current signal decreased slowly, this is because that the excess aptamer immobilized on the electrode surface could hinder the interfacial electron transfer. Thus, 4.0 μmol/L was used. Figure 5c showed the optimized results of the hybridization time between cDNA and aptamer. The current signal increased dramatically in the range of 1.0–2.0 h, and reached a platform when the hybridization time exceeded 2.0 h, indicating the amount of DNA on the electrode surface was saturated. The dependence of the ΔI on the incubation time of OTA was also optimized. As shown in Fig. 5d, it can be seen that the ΔI changed obviously when the incubation time was before 18 min and then changed slowly after 18 min. In order to reduce the detection time for the prepared sensor, 18 min of the incubation time of OTA was used in the further experiments. Figure 5e showed the optimized results of the concentration of CAs-cDNA. The current signal increased dramatically when the concentration of CAs-cDNA increased from 2.0 to 5.0 μmol/L, and reached a maximum of 5.4 μA at 5.0 μmol/L. The current signal decreased when the concentration of CAs-cDNA exceeded 5.0 μmol/L. This was because that the excess CAs-cDNA immobilized on the electrode surface could hinder the interfacial electron transfer.

### Analytical performance of the CAs-cDNA/apt/AuE sensor

**Fig. 6:**
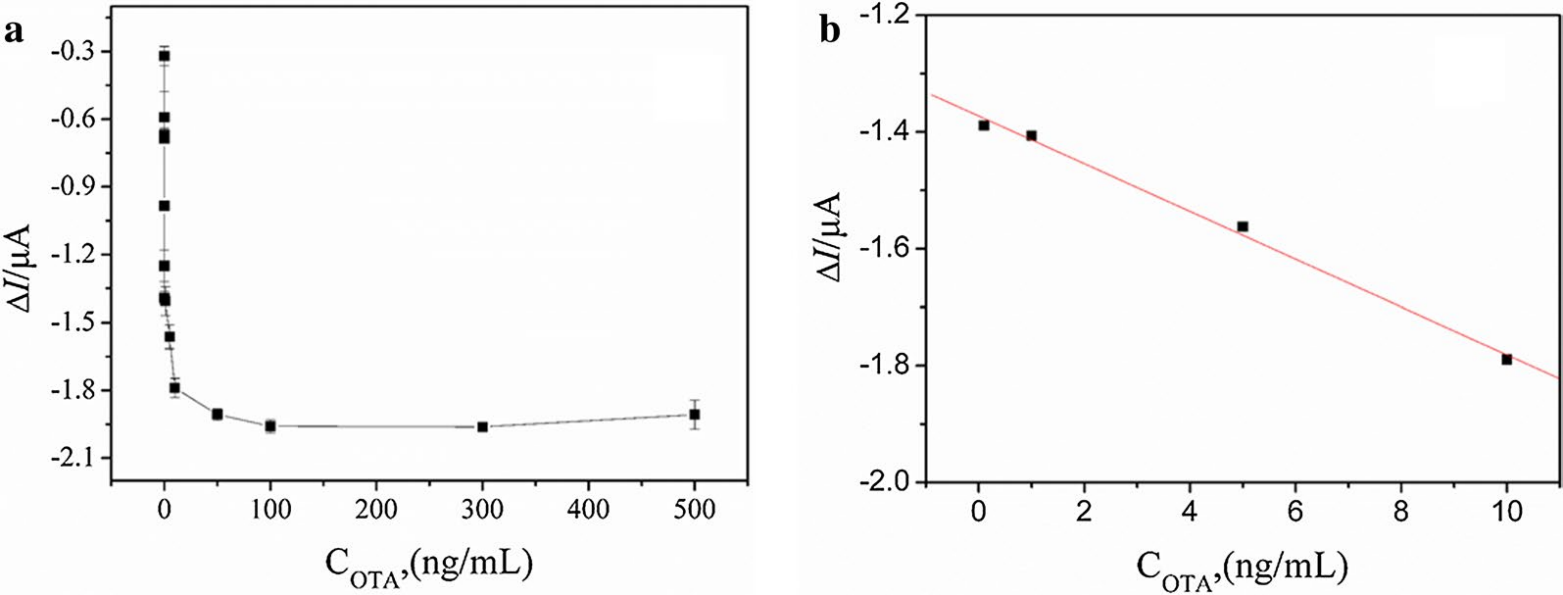
**a** The dependence of ΔI on increasing OTA concentrations. **b** The linear relationship between ΔI and OTA concentrations

Using the optimized parameters, different concentrations of OTA were detected on the CAs-cDNA/apt/AuE sensor. As shown in Fig. 6a, the increase in ΔI was observed upon increasing OTA concentration in the range 1.0 × 10^−4^–500 ng/mL. As shown in Fig. 6b, the ΔI was linearly proportional to OTA concentration in the range of 0.10–10 ng/mL, and the linear regression equation was ΔI = − 0.04x − 1.37 (R^2^ = 0.995). The detection limit of the proposed aptasensor was 1.0 × 10^−4^ ng/mL. Compared with the reported literatures, the CAs-cDNA/apt/AuE sensor was superior to other aptasensors, and the results were shown in Table 1.

**Table 1:** Comparison with other reported aptasensors for OTA detection

Amplified strategy	Detection limit (pg/mL)	Linear range (ng/mL)	References
Enzyme-labeled	1	0.005–10	[27]
AuNPs-GO	0.3	1 × 10^−3^–50	[28]
AuNPs-GO	30	0.1–200	[29]
QDs	0.64	–	[30]
CAs	0.10	0.10–10	This work

### The specificity of the CAs-cDNA/apt/AuE sensor

**Fig. 7:**
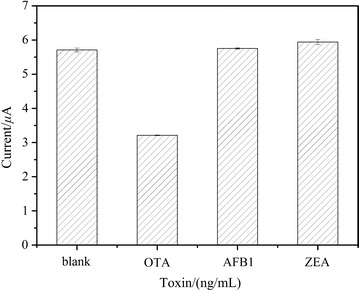
The specificity of the developed aptasensor towards OTA

In order to investigate the specificity of the as-prepared aptasensor, control experiments were performed using AFB1 and ZEA. As shown in Fig. 7, the peak current response from incubation with AFB1 and ZEA did not produce obvious variations compared with that from the blank solution, while induced the great decrease after incubation with OTA. These results demonstrated that the proposed aptasensor had good specificity towards OTA detection.

### Reproducibility and repeatability of the CAs-cDNA/apt/AuE sensor

The reproducibility of the developed CAs-cDNA/apt/AuE sensor was evaluated with inter-assay precision. The five CAs-cDNA/apt/AuE sensors were tested for DPV with same OTA concentration under the same experimental conditions. A relative standard deviation (RSD) of 6.7% was calculated, indicating a good reproducibility of the developed aptasensor. The intra-assay precision of the CAs-cDNA/apt/AuE sensor was evaluated by five repetitive measurements with one electrode and RSD of 7.3% was obtained, indicating that the prepared aptasensor had acceptable repeatability. The prepared CAs-cDNA/apt/AuE was stored at 4 °C when not in use. After a 20-day storage period, the sensor retained 93% of its initial current response, providing the acceptable stability.

### The application of the aptasensor to corn sample

To investigate the actual performance of the developed aptasensor, the OTA concentration in spiked corn sample was examined. As shown in Table 2, the recovery was in range of 86–93% and the average recovery was 89%. This implied that the as-prepared aptasensor had a promising feature for the practical use in corn sample.

**Table 2:** The detection of OTA in the spiked corn sample

Sample	Spiked concentration (ng/mL)	Measure (ΔIp/μA)	Theoretical value C (ng/mL)	Recovery %	Average recovery %
1	10	− 1.77	8.6	86	
2		− 1.78	8.9	89	89
3		− 1.80	9.3	93	

## Conclusions

In this work, a novel CAs-cDNA/apt/AuE sensor was developed to detect OTA using CAs and MB assisted signal amplification. The CAs could load the abundant cDNA and absorb more MB, so the peak current of MB on the CAs-cDNA/apt/AuE sensor was higher than that on the cDNA/apt/AuE sensor. Under the optimized experimental conditions, the developed aptasensor could detect OTA at the level of 1.0 × 10^−4^ ng/mL, and exhibited good specificity against ZEA and AFB1. This sensor was also applied to detect OTA in the spiked corn samples, and an acceptable average recovery of 89% was obtained. By changing the aptamers for different target molecules, this strategy has potential prospect for detecting other targets in the convenient field monitoring.
